# “Business as usual”? Safe-by-Design Vis-à-Vis Proclaimed Safety Cultures in Technology Development for the Bioeconomy

**DOI:** 10.1007/s11948-024-00520-1

**Published:** 2024-11-21

**Authors:** Amalia Kallergi, Lotte Asveld

**Affiliations:** 1 Department of Biotechnology, Section of Biotechnology and Society, Delft University of Technology, Van Der Maasweg 9, 2629 HZ Delft, The Netherlands; 2 Faculty of Science, Institute for Science in Society, Radboud University, Heyendaalseweg 135, 6525 AJ Nijmegen, The Netherlands

**Keywords:** Safe-by-design, Safety culture, Technology development, Bioeconomy

## Abstract

Safe-by-Design (SbD) is a new concept that urges the developers of novel technologies to integrate safety early on in their design process. A SbD approach could—in theory—support the development of safer products and assist a responsible transition to the bioeconomy, via the deployment of safer bio-based and biotechnological alternatives. Despite its prominence in policy discourse, SbD is yet to gain traction in research and innovation practice. In this paper, we examine a frequently stated objection to the initiative of SbD, namely the position that SbD is already common practice in research and industry. We draw upon observations from two case studies: one, a study on the applicability of SbD in the context of bio-based circular materials and, two, a study on stakeholder perceptions of SbD in biotechnology. Interviewed practitioners in both case studies make claims to a strong safety culture in their respective fields and have difficulties differentiating a SbD approach from existing safety practices. Two variations of this argument are discussed: early attentiveness to safety as a strictly formalised practice and early attentiveness as implicit practice. We analyse these perceptions using the theoretical lens of safety culture and contrast them to the aims of SbD. Our analysis indicates that professional identity and professional pride may explain some of the resistance to the initiative of SbD. Nevertheless, SbD could still be advantageous by a) emphasising multidisciplinary approaches to safety and b) offering a (reflective) frame via which implicit attentiveness to safety becomes explicit.

## Introduction

From forever chemicals to microplastics to greenhouse gas emissions, several celebrated and in times lifesaving innovations turned out to have significant adverse effects. Could such negative impacts have been anticipated? Could they have been prevented? Retrospective speculation is a perilous activity, yet it is plausible that at least some adverse effects of technological innovations could have been avoided, if we were to look carefully, closely and on time. In an effort to prevent past regrets and to minimise the adverse effects of novel technologies, the concept of “Safe-by-Design” (SbD) advocates an increased attentiveness to safety at early stages of technology development (Ministry of Infrastructure & Water Management, 2019; OECD, 2020). In accordance with the proverbial wisdom that the prevention of risks is better than their management, SbD is a risk management approach that urges developers to consider safety early on in their design process. The assumption is that early and increased attention to safety will lead to appropriate safety-motivated design choices that will—in turn—generate safer products.

A transition to the bioeconomy provides a promising path when dealing with the dire environmental challenges that our societies face. Bio-based modes of production can potentially reduce the environmental impacts of our modern lifestyles and can limit stress on finite planetary resources. Biotechnological and bio-based routes of production are often more sustainable than their chemical counterparts while bio-based products can demonstrate environmentally desirable properties such as biodegradability. Despite their potential, no technological solution can be ethically permissible and societally desirable unless it is acceptably safe. A SbD approach could thus be of relevance to biotechnologists and engineers working on a responsible transition to the bioeconomy, by aiding the development and deployment of safer bio-based and biotechnological alternatives.

While the aims and rationale behind SbD are unquestionably noble, SbD can only be impactful if embraced by practitioners. As shown in the next section, the concept has a relatively recent history while formal implementations of SbD for the bioeconomy are uncommon. For SbD to gain traction in academic and industrial practice, practitioners must accept it as a worthwhile development. Presently, this is not a given (Asin-Garcia et al., 2023; Bouchaut & Asveld, 2020). Understanding how SbD is perceived by practitioners can thus inform the future of this initiative. In this paper, we examine a frequently stated objection to the initiative of SbD, namely the position that SbD is already common practice in research and industry. Our analysis draws upon observations from two case studies: one, a study on the applicability of SbD in the context of bio-based circular materials and, two, a study on stakeholder perceptions of SbD in biotechnology. We detect a recurring argument in both case studies which we analyse using the theoretical lens of safety culture. Loosely understood as “the way we do things around here” (Guldenmund, 2000; Hopkins, 2018), the notion of safety culture is a useful analytical tool that enables us to unravel practitioners’ perceptions about their domains of practice, to capture relevant particularities of each domain, and to elucidate barriers and opportunities to the initiative of SbD.

## Theoretical Framework

### SbD as Responsible Innovation

SbD is a new concept that urges the developers of novel technologies to integrate safety early on in their design process. It represents a pre-emptive approach to risk management that emphasises the prevention of risks versus their management (Ministry of Infrastructure & Water Management, 2019; OECD, 2020; Robaey, 2018; van de Poel & Robaey, 2017). SbD calls attention to the negative impacts of an innovation all across its lifecycle and advocates that (some of) these negative impacts can be minimised if considered early on in the development process. In effect, SbD promotes a shift of responsibility to the research and development (R&D) phases (Robaey et al., 2018) and to researchers and developers, who should anticipate risks and uncertainties early on and respond accordingly. A SbD approach could—in theory—support the development of safer products and assist a responsible transition to the bioeconomy, via the deployment of safer bio-based and biotechnological alternatives.

SbD shares conceptual similarities with various engineering traditions for increasing safety, such as the notion of inherent safety (Amyotte et al., 2009; Kletz & Amyotte, 1998). Nonetheless, the term SbD was coined in the context of nanotechnology (Kelty, 2009) and in response to early concerns about the unknown risks of nanomaterials. In this context, SbD emerged as an approach for researchers and developers to *proactively* improve the safety of their nanomaterials, by making safety-informed choices at early stages of their investigation. Similar understandings of SbD can be traced in other emerging technologies, such as synthetic biology. For example, synthetic biologists develop dedicated biosafety tools that pre-emptively address the risks and uncertainties of synthetic biology (Asin-Garcia et al., 2020; Moe-Behrens et al., 2013). The proactive and pre-emptive aspects of SbD reflect parallel developments in policy and innovation, such as the Responsible Research and Innovation (RRI) framework (von Schomberg, 2013). RRI calls for innovations developed *for* and *with* society, via anticipatory, reflective, responsive and inclusive processes (Stilgoe et al., 2013). SbD can be thus understood as an instance of an RRI methodology that aspires to innovate in line with the societal value of safety.

Various engineering fields implement the mandate of SbD in different ways and via different principles (van Gelder et al., 2021). To date, nanotechnology offers the most concrete actualisation of SbD into a practical methodology; an exhaustive overview of (EU-funded) frameworks and toolkits for the development of safer nanomaterials is provided by Krans et al. (2021). While typically associated with emerging technologies, SbD can be of relevance to the development of any product. For example, the Chemicals Strategy for Sustainability (CSS) (European Commission, 2020) strongly institutionalises the (closely related) concept of Safe-and-Sustainable-by-Design (SSbD) as the desired modus operandi for (future) chemical industries. This paper focuses on two domains of practice pertinent to the bioeconomy, namely biotechnology (specifically: white and green biotechnology) and bio-based material production.

### Safety Culture

If it were one thing that safety culture scholars share, this would be a discontent with the numerous interpretations, uses and misuses of the term. Originating from an interest to understand and prevent accidents in the workplace (Dekker, 2019), the notion of safety culture gained significant attention in the field of organisational studies. Definitions of safety culture abound but all seem to share an interest in a group’s beliefs (values, attitudes, norms) and practices in relation to safety (Cooper, 2000; Guldenmund, 2000; Wiegmann et al., 2004). In the context of this study (i.e. technology development), we will use the term “safety culture” to refer to safety-related beliefs and practices held by practitioners who engage with R&D activities in an institutional setting. In academic settings, this predominantly refers to researchers, engineers, and developers. In industrial settings, this may refer to both scientific and managerial staff. In effect, safety culture stands for what has been previously described as “the way we do things around here” (Guldenmund, 2000; Hopkins, 2018). In the context of this study, the “we” is practitioners involved with technology development and the “here” a company or a research group.

Understandings of safety culture (or rather, of culture in general) tend to fall under two camps (Dekker, 2019; Glendon & Stanton, 2000; Guldenmund, 2015). The interpretivist one assumes that culture *is* (and exists regardless) while the functionalist one assumes that a culture is *had* (and can be managed). We align with the former and with anthropological or interpretive approaches on safety culture that conceptualise culture as an emergent phenomenon of a group (Haukelid, 2008). It follows that a safety culture exists regardless of its success, quality, or maturity. In this case, the term should *not* designate a value judgement, nor should it suggest that safety is successfully integrated in the given setting. When necessary, we will use the qualifier “strong” to describe a safety culture characterised by a strong commitment to safety.

Traditionally, safety culture is examined in relation to occupational safety and the prevention of accidents that affect both workers and surrounding communities. In the context of technology development, this could be comparable to lab safety, i.e. the safety of the environment where R&D activities take place. Nevertheless, our conversations with practitioners were part of an effort to understand the meaning and relevance of SbD. Emphasis was therefore on the safety of the future technological product, including its future production, use and disposal. It follows that “safety” in our use of the term “safety culture” refers primarily to the safety of a novel product, not of the environment where this product is currently researched.^1^ In recent literature, this is sometimes coined as “product safety culture” (Suhanyiova et al., 2021).

**Fig. 1:**
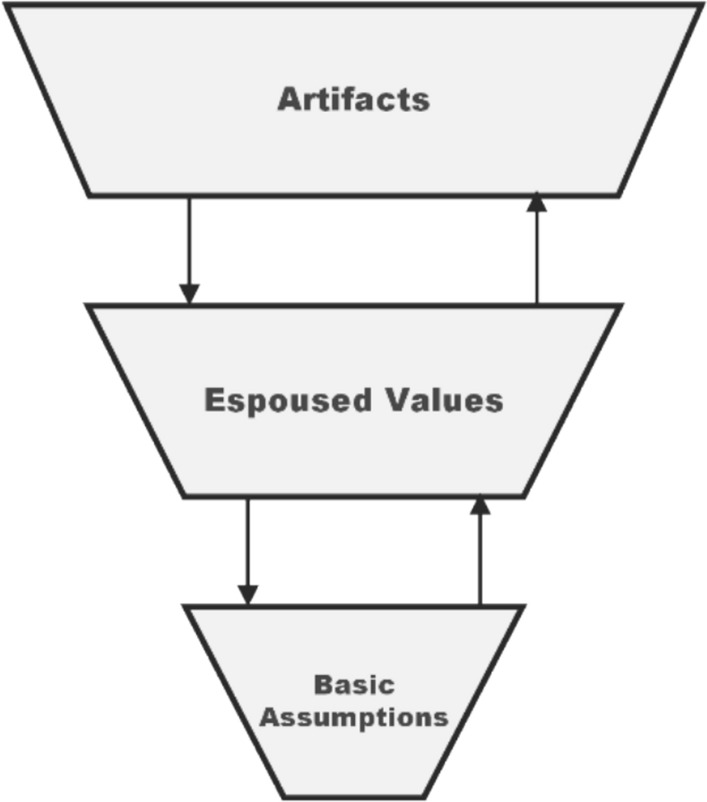
Schein’s levels of culture. Figure adapted from Guldenmund (2018)

Schein’s classic three-layer model offers an analytical tool for the study of organisational cultures, of which safety cultures are a subset. According to Schein (2010), an organisational culture can be studied at the level of artefacts, espoused values, and basic assumptions (cf. Figure 1). Artefacts are the most readily accessible to a researcher, while deeply rooted assumptions are the hardest to capture. For Schein, artefacts include not only physical objects (e.g. a safety poster) but also processes (e.g. a safety protocol) as well as the observable behaviour that these structures result in. Espoused values, on the other hand, refer to the articulated beliefs, values, and ideologies of an organisation; these may or may not correspond with the layers above and below. Finally, basic assumptions are what the members of a group take for granted. These deeper assumptions are often unconscious and unarticulated but do affect the behaviour of the group.

**Fig. 2:**
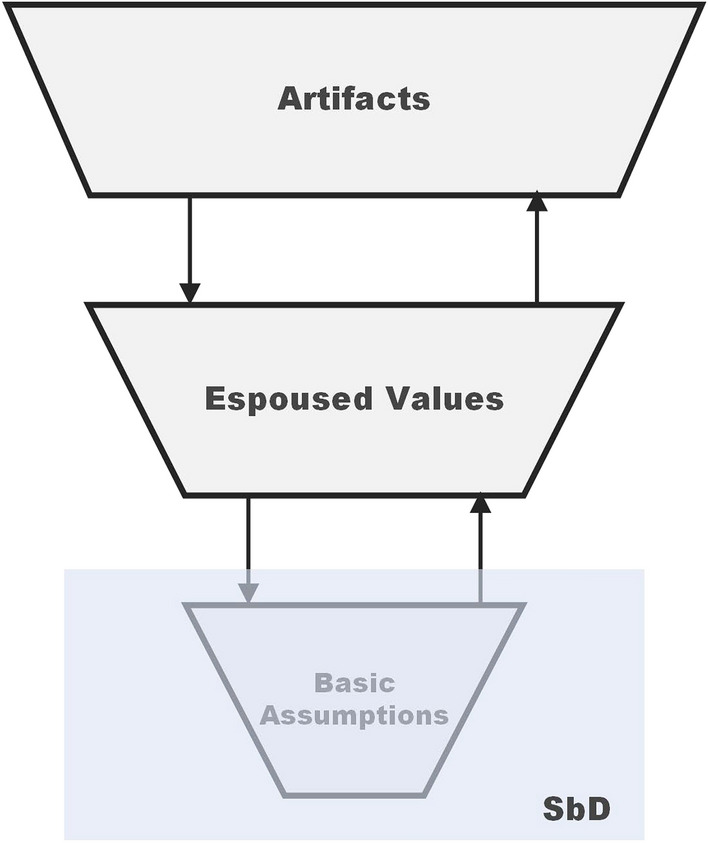
Schein’s model and SbD. SbD aspires to affect professional practice (i.e. what developers do) by changing what developers think about safety, e.g. that safety is as important as functional requirements or that safety is something to be considered already at early stages. Figure adapted from Guldenmund (2018)

In this work, we employ Schein’s model purely as an analytical tool: we use it to formalise practitioners’ perceptions and contrast them to the aims of SbD. Note that SbD assumes that safer professional practices and behaviours emerge when safety is prioritised as a core value, i.e. when (early) attentiveness to safety becomes a taken-for-granted assumption (cf. Figure 2).

## Methodology

This work uses the theoretic lens of safety culture to interpret empirical findings from two subsequent case studies conducted in the Netherlands, in the period between December 2020 and December 2021.

### Case Studies

Case study A (Kallergi & Asveld, 2021a) examined the applicability of SbD in the context of the circular economy. Specifically, it questioned whether risks associated with novel circular materials could be minimised via a SbD approach. We conducted semi-structured interviews with the members of a research consortium tasked with developing a novel circular biocomposite.^2^ Participants were asked about relevant risks and uncertainties and were invited to identify or brainstorm suitable SbD actions. These interviews captured both the realities of the development process and the practitioners’ understanding of how SbD could be operationalised. All interviewees (n = 8) were internal to the consortium, with academic and industrial partners equally represented. All but one interviewee were researchers or professionals of medium to high seniority, with extensive experience in the field of bio-based and circular materials. Interviews were conducted online, videorecorded, transcribed, anonymised, and coded using a hybrid approach in *Atlas.ti*. In parallel, we attended internal meetings held monthly by the consortium and observed issues of relevance as the project evolved in time. These ethnographic observations, captured in meeting notes, complemented our findings with practical examples.

Case study B (Kallergi & Asveld, 2021b) examined how relevant stakeholders perceive the concept of SbD in biotechnology. We conducted semi-structured interviews with representatives of various stakeholder groups (civil society, industry, academia). Our sample included interviewees from diverse backgrounds and professional interests, many of whom were unfamiliar with the concept of SbD. Of relevance to the present work are the responses of industry representatives (n = 6) expected to implement SbD in their practice. Among the questions asked were general questions about SbD (e.g. prior knowledge; first impression/own definition; risks and benefits) as well as questions about different types of SbD actions. Interviews were conducted online and videorecorded. Video recordings and notes were used to write anonymised summaries for each interview, which were then inspected for common themes.

### The Present Study

While conducted independently and with separate research aims, both case studies demonstrated noticeable similarities in the ways practitioners reasoned over SbD. Specifically, we detect a recurring argument brought up by interviewees, namely that SbD is already common practice in their respective domain. Informed by codes and memos that emerged independently in the two case studies, this work takes a next interpretive step to offer a synthesis and re-interpretation of our previous findings. The present synthesis represents our reflection as researchers immersed in both case studies; due to the heterogeneity of study designs, we consciously refrained from pooling and re-coding the collected data.

Methodologically, we inspect the identified arguments through the theoretical lens of safety culture with the objective to explicate their implications for SbD. The practitioners’ arguments, while verbal, imply a certain way of “doing things”, i.e. they *proclaim* a safety culture. In our analysis, we use Schein’s model^3^ to reconstruct these proclaimed safety cultures. Obviously, these reconstructions tell us little about the *actual* safety cultures in the corresponding domains; they do, however, tell us plenty about how practitioners *perceive* their own domains of practice. These perceptions are consequential for SbD which is envisioned to be voluntarily embraced by practitioners. Moreover, professional identities are partially formed via discursive processes, so attention is due to how practitioners speak about themselves and their professional domains. Dominant ways of thinking about safety practices in one’s domain may prevent practitioners from attending to new types of challenges or from recognizing new opportunities for action.

## “We Are Already Doing it”: A Recurring Argument

A typical response by practitioners in both case studies is the position that SbD is already common practice in their respective domains. Engineers and biotechnologists stress that (early) attentiveness to safety is an indispensable part of their work, that safety is commonly understood as important, and that early attentiveness to safety is something they cannot afford to omit. This position takes different shape depending on the context of each interviewee, but the essence of the argument is that the approach advocated by SbD is something that practitioners are *already doing*, either explicitly or implicitly.

### Early Safety Attentiveness as a Strictly Formalised Practice

Practitioners in case study B (biotechnology) suggest that early attentiveness to safety is part of a *strictly formalised* and streamlined practice. They understand themselves (and their colleagues) as adhering to a SbD-like approach by default, as they engage in their regular professional practice. For some, early attentiveness to safety is necessitated by the strict regulatory regime currently in force in the EU. For example, a requirement for early registration means that safety issues must be considered and documented early. These conditions may be specific to biotechnology and may dictate an explicit discourse on safety across the organisation. Other responders note that early attentiveness to safety is necessitated by (market) logic: No company is willing to waste time and resources to develop a product that will be deemed unsafe at the end. As remarked by one interviewee, a safe product can only be achieved “by design” and careful planning; safe products do not just happen by accident or coincidence. Early consideration of safety aspects, training, good documentation, and regular checkpoints are some of the internal structures referred to by practitioners.

Early safety attentiveness as a strictly formalised practice was mostly observed in case study B (biotechnology), i.e. a domain that is strictly regulated. A summary of the EU regulatory framework on GMOs is provided by Bruetschy (2019). In the Dutch context, Bouchaut and Asveld (2021) showcase a strict safety regime for biotechnology. This regulatory context should not be misunderstood for actual practice, nor does it automatically imply that existing practices are equivalent to SbD. However, the unique regulatory circumstances of biotechnology in the EU lend partial support to the argument of strictly formalised practice. Contrarily, practitioners in case study A (novel materials) struggled with a regulatory system that is not yet equipped to deal with novel circular materials. The reasoning that early safety attentiveness makes commercial sense was iterated by two participants in case study A too.

### Early Safety Attentiveness as Implicit Practice

Several responses from case study A (novel materials) suggest early attentiveness to safety as an *implicit* practice. Practitioners understand themselves (and their colleagues) as adhering to a SbD-like approach, albeit in an implicit manner. This applies both to general comments about the importance of safety and to specific safety-related actions. For example, participants comment that safety is “always in the back of our heads” (in a participant’s own words). Naturally, their emphasis is on “always” and on safety attentiveness as a form of instinct associated with good professional practice. Yet, these comments also suggest a lack of explicit discourses around safety. Specific safety-related actions or decisions are also reported as undertaken implicitly. Consider as an example the choice of additives in the recipe of the biocomposite. The commitment to use natural additives was highlighted as a SbD-like action that preventively and proactively enhances the safety of the final product. At the same time, it was described as an implicit choice made at earlier stages of the project.

Early safety attentiveness as implicit practice was mostly observed in case study A (novel materials), i.e. a case that approximates applied research. This case study also produced a list of already implemented (design) actions recognised by developers as pertinent to SbD (e.g. choice of additives, high temperature); this practical overlap lends indirect support to the argument of implicit practice. In case study B (biotechnology), a latent safety instinct was noted in the context of plant breeding: it was remarked that safety expertise is not always easily communicable and that breeders in small-scale enterprises often use their (tacit) taxonomic knowledge and field^4^ experiences when making safety-enhancing choices.

### A Strong Safety Culture?

Both flavours of the “*we are already doing it*” argument make claims to a *strong* safety culture in which safety is collectively acknowledged as an important and shared value. The individual researchers and developers, the companies or organisations in which these practitioners work, and, in times, the entire sectors of biotechnology and engineering are referred to as devoted to the value of safety. Direct or indirect references to (rival) organisations (or countries) with a *poor* safety culture are occasionally made, but the value of safety is generally acknowledged as pivotal. Moreover, responders are confident that this shared attitude results in safety-motivated choices during product development. In other words, the proclaimed safety culture is understood to guide the actions of individuals operating in the corresponding organisational unit.

**Fig. 3:**
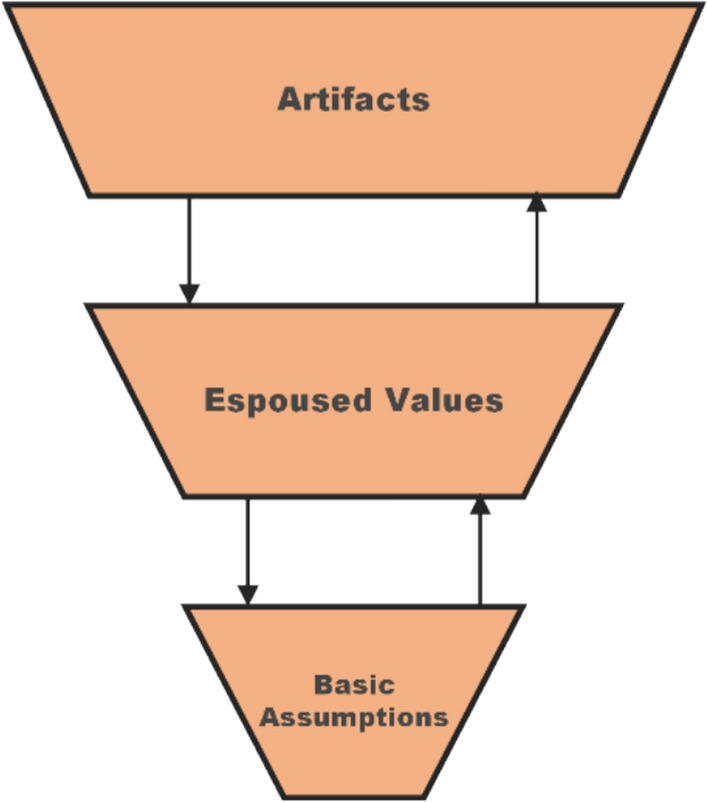
Early safety attentiveness as a strictly formalised practice. The safety culture implied by this argument would manifest at all levels of Schein’s model, with artefacts having a structural connection to basic assumptions. Figure adapted from Guldenmund (2018)

**Fig. 4:**
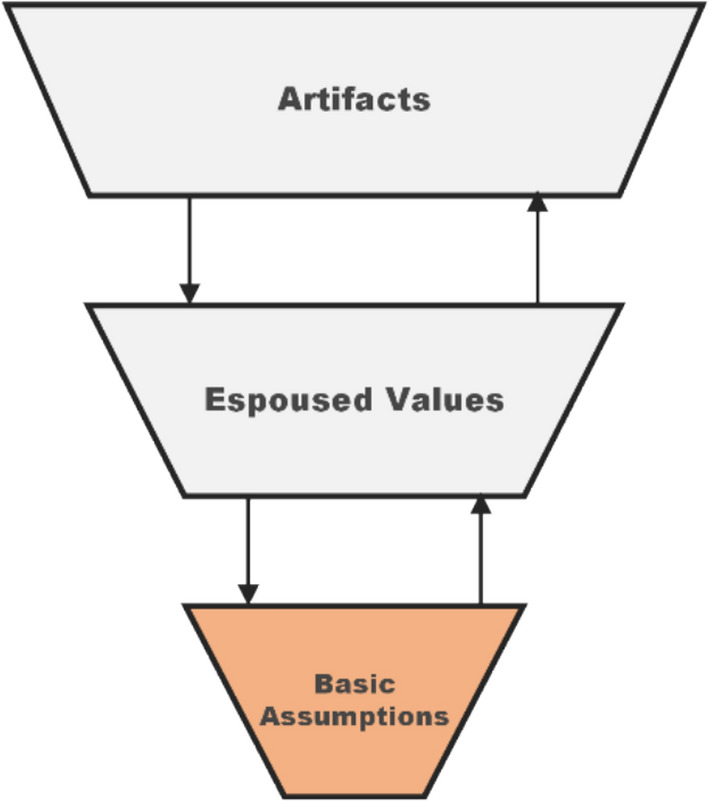
Early safety attentiveness as implicit practice. The safety culture implied by this argument would manifest at the deeper level of Schein’s model but would remain unarticulated and invisible. Figure adapted from Guldenmund (2018)

Seen through the lens of safety culture, “*early safety attentiveness as a strictly formalised practice*” shows a strong manifestation at the level of artefacts, such as rigorous procedures, protocols, and paperwork to safeguard safety. Contrary to what is usually said about artefacts, i.e. that they may be only superficial signs of safety beliefs, participants refer to these structures as evidence of good professional practice and of a strong commitment to product safety (cf. Figure 3). Naturally, one can question whether compliance to external constraints (e.g. regulatory demands) qualifies as a strong safety culture. Yet, responders seem to take pride in this formalisation of safety behaviours in biotechnology, which they often describe as superior to what is achieved or required in other disciplines.^5^ “*Early safety attentiveness as implicit practice*”, on the other hand, lacks such a manifestation in overt artefacts. Nevertheless, it makes equally strong claims to a commitment to safety and to a safety culture that manifests at the deeper level of assumptions and held beliefs (cf. Figure 4). It implies a deeply rooted conviction, a second nature and an instinct that defines the profession of researchers and engineers.

## *SbD*: Friend or Foe?

Having reconstructed the safety cultures implied in the practitioners’ arguments, let us now examine them in direct comparison to the aims of SbD. As explained, we do not wish to question whether the corresponding sectors operate as claimed. Rather, we focus on the participants’ perceptions of their own domains and on the implications of these views for SbD.

### *SbD* Offends Professional Sensibilities…

The first insight of our analysis is an unexplored link between SbD and the professional identity of engineers. This link exposes a root source of discontent with SbD and points to a need to frame SbD as a multidisciplinary approach that invites a wider set of perspectives.

Both flavours of the “*we are already doing it*” argument can be interpreted as a proclamation of virtue (we *do* have a strong safety culture; we *do* care about safety). At the same time, they are also proclamations of (mild or strong) annoyance with SbD, which is perceived as redundant and unsolicited. Either way one can trace an element of professional pride or professional self-realisation associated with the value of safety. In the case of “*strictly formalised practice”,* the referred practices are part of a (historically) long trajectory to satisfy demands of a strict regulatory framework, of a predominantly precautionary regime and of a distrustful public. Against this backdrop, early attentiveness to safety is core to actualizing the field’s professionalism and responsible conduct. It should thus come as no surprise that any insinuation of the opposite (as, arguably, SbD may imply) is met with hostility. The situation is similar with “*implicit practice”*. This argument positions safety at the level of basic assumptions that are part of an engineer’s professional skillset, sensibilities, and role responsibility.^6^ By urging engineers to think differently (more, earlier, etc.) about safety, SbD directly offends the professional identity of practising engineers.

The proposition that SbD offends professional sensibilities will need to be tested empirically. It will also need to be examined in comparison to earlier shifts on what it means to be a good engineer: how do new (societal) requirements become negotiated into the professional identity of engineers? Meanwhile, it is conceivable that SbD discourse is misplacing its focus. Instead of urging practitioners to think differently about safety, SbD could capitalise on professional pride and existing capabilities to collaboratively devise pragmatic ways for practitioners to actualise their professional responsibility. When applicable, this support should capture tacit, unarticulated safety-related knowledge. Moreover, as we argue elsewhere (Kallergi & Asveld, 2021a), a more pragmatic SbD approach should also support coordination and information exchange *across* the value chain, i.e. beyond the confines of one professional group.

Obviously, treating safety as part of an engineer’s role responsibility is assumed as positive, i.e. as contributing to safer products. After all, SbD aspires to instil a similarly fundamental commitment to safety. Yet, this assumption begs the question: *should* (the quest for) safety be conceptualised as part of an engineer’s role responsibility? Objections may be raised on grounds of self- regulation: can safety be sufficiently guaranteed when engineers are made responsible for the safety of their own designs? We deem such concerns secondary: if SbD is a means to an end (i.e. safer products), it can be assumed that the final product will be still rigorously regulated. Our concerns revolve around a different consequence, namely the risk that developers become the *sole* experts on safety. This would directly deny safety as a sociocultural construct that demands consideration from a multiplicity of perspectives (Schwarz-Plaschg et al., 2017). Here lies a direct opportunity for SbD: To support the development of safer products via the inclusion of stakeholders and multiple perspectives. As argued by Bouchaut and Asveld (2021), openness to all stakeholders is a necessary condition for responsible learning about safety and one that can be met via a SbD approach.

### …but Implicit Practice is a Source of Ambiguity

Our second insight arises from the limitations of “*implicit practice”* and points to a role for SbD in overcoming these limitations. References to implicit practice acknowledge that basic assumptions about safety can guide behaviour positively. Yet, regardless of how genuine or strong these safety beliefs are, implicit practice quickly becomes a source of ambiguity that hinders honest efforts to design for safety. Moreover, it constitutes a missed opportunity for sharing safety-related knowledge.

A primary function of culture is to support the members of a group to respond to key situations. Shared values can guide us and teach us how to cope but, for this, shared values should be accessible and in a conscious plane. In the case of early attentiveness to safety as implicit practice, the (strong but unarticulated) basic assumptions offer little guidance when navigating everyday situations. An engineer’s instinct may be to always enhance safety, but developers are continuously faced with everyday decisions, often under time pressure or a pressure to achieve results (e.g. reach a minimum viable product). More importantly, when early attentiveness to safety remains implicit, conflicts between co-existing values such as safety, sustainability, and innovation are likely to emerge. Let us illustrate this point with an ethnographic observation from case study A (novel materials).

Implicit attention to safety is often associated with a general desire to be socially responsible. In case study A, all developers and researchers involved in the consortium understood their project as an effort to provide safer alternatives to existing materials. Their efforts also contribute to less detrimental modes of production and consumption by developing more sustainable materials. Conspicuously, the *implicit* choice to use only natural additives is a strategy that serves both goals. As a result, the rationale behind the additives used becomes quickly blurry: are natural additives chosen because they are non-toxic or because they are more sustainable? What happens when these two aims contradict each other? During the project, an LCA analysis indicated that a particular additive should be replaced by a fossil-based alternative of better environmental performance. This suggestion caused internal conflicts in the consortium, with some being uncomfortable with the switch but unable to fully justify their unease. Because the choice of additives was an *implicit* safety-motivated choice, decision-making eventually lost its safety orientation while the range of candidate additives became internally inconsistent.

Here lies another opportunity for SbD: to explicate and maintain in an accessible plane what is now implicit. Consciously adopting a SbD frame could, for example, help systematise safety efforts and prevent slips and omissions. Moreover, a SbD approach should encourage ongoing reflection: how do our basic assumptions about the importance of safety manifest in practice? What other values may affect our decision-making? Reflective exercises can also aid developers in crosschecking the power of their basic assumptions: do they lead to safety-enhancing actions or are they only wishful thinking? Finally, as mentioned in the previous subsection, making the implicit explicit can benefit others by sharing and formalising safety-related know-how.

## Discussion

### Study Limitations

This contribution synthesised and re-interpreted empirical findings from two case studies. This synthesis derived from our continuous reflection over the findings of both case studies. This approach has obvious limitations compared to a dedicated empirical study with a predefined focus on argumentation. Our proposed model (i.e. main argument and its two flavours) fits our joint observations best but might not necessarily reflect the *conscious* rhetoric strategies of our participants. Nevertheless, both flavours of the argument are directly traceable in our data and form a justifiable abstraction of the participants’ views towards SbD and their own domains of practice.

Together, our case studies represent the views of 14 expert practitioners: 8 practitioners active in the field of bio-based and circular materials and 6 representatives of biotech companies or professional associations, the latter being also the spokespersons of their sectors. Nevertheless, it is possible that the views collected are not representative: they may be specific to the individuals interviewed and may reflect a minority position in each sector. As with any interview study, responses may be biased (e.g. politically correct or strategic) and may not correspond with actual practice. In safety culture terminology, our observations were collected at the level of espoused values (i.e. beliefs articulated verbally) which should not be taken at face value. Indications that support the practitioners’ arguments were found; this study, however, makes no claims about actual safety cultures as our focus was exclusively on the *perceptions* of practitioners. Studies to contrast what is being said and what is being done as well as studies to examine coexisting basic assumptions that may indirectly affect safety behaviours can better capture the safety status quo of the respective domains.

Our interviews broached the subject of SbD by focusing on technology development in R&D settings (in either academia or industry) and by highlighting complementary aspects of a SbD approach. Emphasis was on SbD as early action, on SbD as mitigating responsibility to developers, and on SbD as acknowledging safety as a core value. It should be noted that the domains under examination also lack a more systematised implementation of SbD, as compared to, e.g. nanotechnology. These elements may have contributed to our participants’ reluctance to distinguish SbD from existing practices. An element of SbD that may be more novel for practitioners is the consideration of risks *throughout* the product lifecycle, especially risks at the end-of -life of the product. Likewise, our case studies took place in the Netherlands and, as such, reflect a temporary and culturally bound view of the engineering profession. As shown by Downey et al. (2007), the identity of engineers (and its relationship to ethical responsibilities) can vary considerably across national contexts.

### Safety Culture as an Analytical Tool

The main contribution of this paper lies in explicating the implications of certain views and arguments for the future of SbD. In this respect, the introduction of the theoretical lens of safety culture was particularly instructive, despite it being an admittedly ill-defined and problematic concept (Guldenmund, 2000; Pidgeon, 1998; Silbey, 2009). Using Schein’s model as an analytical tool allowed us to pinpoint a root cause of the practitioners’ discontent with SbD and to identify possible ways forwards for the initiative of SbD. These insights were reached *thanks to* the theoretical exercise of reconstructing the proclaimed safety cultures. Being based on the perceptions of practitioners, our insights are also unaffected by the validity of these perceptions. Effectively, we have taken an approach that is attentive to the attitudinal—even affective—dimensions of negotiating the role of SbD in the work floor. Insights gained can inform better communications about SbD as well as better practical implementations.

This work conceptualised safety culture as something that is/emerges rather than something that is managed. We have thus used the notion of safety culture as an analytical tool only. Those interested in operationalizing SbD may benefit from functionalist approaches to safety culture, specifically efforts to improve the safety culture (or climate) of an organisation. Wiegmann et al. (2004) report five indicators of safety culture, namely organisational commitment, management involvement, employee empowerment, reward systems, and reporting systems. Some of these indicators may be applicable in the context of technology development too. In our research (Kallergi & Asveld, 2021a, 2021b), we have identified the need for SbD actions at the organisational level, including but not limited to education, interdisciplinary teams, and freedom to raise safety concerns.

## Conclusion

This paper analysed a recurring response to SbD raised by practitioners engaging with technology development for the bioeconomy. Informed by empirical findings from two case studies and using Shein's model as an analytical tool, we identified points of friction and possible ways forward for SbD. The “*we are already doing it*” argument implies that biotechnologists and engineers are already engaging in some form of SbD. They do so either explicitly, i.e. via strictly formalised procedures, or implicitly, i.e. as part of good engineering practice. These views (and the safety cultures they proclaim) affect how SbD is evaluated by those called to embrace SbD in practice. Resistance to SbD can be easily explained both pragmatically and emotionally if a call to SbD is at odds with (and even offensive to) the way practitioners perceive themselves and their profession. Nevertheless, our analysis reveals that SbD can still be advantageous by a) emphasising multidisciplinary approaches to safety and b) offering a (reflective) frame via which implicit attentiveness to safety becomes explicit and, thus, accessible and negotiable. Eventually, this work reminds us that the initiative of SbD does not happen in a vacuum. It occurs in a discursive space that is shaped by perceptions, existing practices, ongoing debates, and the histories of the corresponding fields. Any attempt to introduce SbD in a domain of practice will need to pay close attention to these contextual factors.

## Footnotes

1 Naturally, safety-related actions meant to improve the safety of a future product can be beneficial for the safety of the researcher involved in its development. Consider as an example the use of a non-pathogenic organism and the benefits of this choice for both researchers and future users.

2 Composites are reinforced plastics that consist of two primary components, i.e. a polymer and a net of reinforcement fibres. In a biocomposite, at least one of the two components is of natural origin, typically a combination of a synthetic polymer with natural (plant, animal, or waste) fibres (Zini & Scandola, 2011).

3 Technically speaking, Schein’s model is a model of organisational culture *applied to* safety culture (cf. sub-Sect. "Safety culture"). Due to the model’s popularity in safety culture studies, we will not further mark this distinction. We thank an anonymous reviewer for highlighting this nuance.

4 The word “field” is used here literally to refer to a field of agricultural land.

5 In the words of one participant, biotechnologists “are not cowboys”, a reference to innovation domains with less mature regulatory frameworks.

6 Consider as an example the code of ethics for engineers by the National Society of Professional Engineers (NSPE, 2019) stating that “engineers shall hold paramount the safety, health, and welfare of the public.”.
